# Addressing Future Changes in Communication Using Hypothetical Scenarios With People With Primary Progressive Aphasia and Care Partners: A Conversation Analytic Study

**DOI:** 10.1111/1460-6984.70235

**Published:** 2026-04-05

**Authors:** Winsnes Ingvild, Halvorsen Mia, Krogh Elise, Volkmer Anna

**Affiliations:** ^1^ Department of Linguistics and Scandinavian Studies University of Oslo Oslo Norway; ^2^ The Faculty of Education and Arts Nord University, Norway; ^3^ Division of Psychology and Language Science University College London London UK

**Keywords:** advance care planning, conversation analysis, dementia, primary progressive aphasia

## Abstract

**Background:**

People with primary progressive aphasia (PPA) will experience a decline in language and cognitive function, and behavioural changes are not uncommon. Decline in everyday skills has been reported and becomes more pronounced over time. Previous research has shown that people with PPA and their care partners (CPs) want to address future changes. Addressing future changes and future planning is highlighted in guidelines, such as the National Institute for Health and Care Excellence's (NICE) guidelines on Decision‐making and mental capacity. The Better Conversations with PPA is a manualised program, where planning for future changes is addressed.

**Aims:**

This study aimed to explore how the topic of planning for the future in Better Conversations with PPA is undertaken in conversations during the treatment sessions.

**Methods:**

Conversation analysis (CA) is highlighted as the “gold standard” for analysing recorded healthcare communication. Using applied CA, a method to systematically study social interaction, we analysed video recordings from four dyads, comprising a person with PPA and a care partner, participating in the Better Conversations with PPA program. The sessions are led by a speech and language therapist.

**Outcome and results:**

Our analysis shows that the structure of the conversations about the future is closely aligned with the structure of the Better Conversations with PPA program session plan. The speech and language therapist creates several opportunities for the dyads to engage in conversations about the future, using hypothetical questions and scenarios, creating a conversation environment where future changes can be addressed without the dyads having to relate this to themselves. Thereby orienting to the delicacy of the topic.

**Conclusion:**

The Better Conversations with PPA program provides a promising framework for addressing future changes. The use of hypotheticals seems to be a useful strategy to promote sensitive conversations about future changes.

**WHAT THIS PAPER ADDS:**

*What is already known on this subject*

- Primary progressive aphasia is a degenerative disease. Addressing future changes and future planning is highlighted in guidelines, such as the National Institute for Health and Care Excellence's guidelines on Decision‐making and mental capacity. The Better Conversations with Primary Progressive Aphasia is a manualised program, where planning for future changes is addressed. However, less is known about how the topic is treated by people with primary progressive aphasia, care partners, and speech and language therapists.

*What this study adds to existing knowledge*

- Using conversation analysis to explore the topic of future planning in the Better Conversations with Primary Progressive Aphasia program, we found that the structure of these conversations is closely aligned with the structure of the program. Hypothetical questions and scenarios seem to be particularly powerful in addressing future changes and planning for these.

*What are the potential or actual clinical implications of this work?*

- The Better Conversations with Primary Progressive Aphasia might be used as a framework to address future planning. Hypotheticals seem to be a useful strategy.

## 1 Introduction

Globally, more than 57 million people are living with dementia, and this is estimated to increase to more than 152 million by 2050 (Nichols et al. 2022). Primary progressive aphasia (PPA) refers to a group of language‐led dementias (Marshall et al. 2018). A conservative estimate of the prevalence of PPA is 3–4 cases per 100,000 (Coyle‐Gilchrist et al. 2016; Marshall et al. 2018). Difficulties with speech and language are the first and most prominent symptom in PPA (Gorno‐Tempini et al. 2011). These language difficulties will become more pronounced over time, where both language production and comprehension are affected, and may lead to profound global aphasia, and in many cases of PPA, mutism (Ulugut et al. 2022). Additional deficits in other cognitive domains (e.g., memory and orientation problems) and behavioural changes (e.g., stereotypical behaviour and apathy) have been reported in the early stages, becoming more significant over time (Foxe et al. 2022; Ulugut et al. 2022). Decline in everyday skills (e.g., handling money) and self‐care (e.g., hygiene) become more pronounced over time, and, together with motivation, have been reported to be the areas with the greatest annual rate of change in PPA (Foxe et al. 2022).

People with PPA (PwPPA) and their families have expressed a need for information about the future, to enable them to plan and make decisions in advance. In a consensus study which included 91 care partners (CPs) of PwPPA from across 15 countries, participant CPs identified ‘Talking about the future’ as the fifth top priority when asked what they wanted to change about their lives with PPA (Volkmer et al. 2024). ‘Getting more information about PPA and the stages’ was rated 10^th^ by CPs, yet PwPPA did not identify this as a priority. Importantly, in a qualitative interview study with PwPPA, Davies et al. (2025) found that whilst PwPPA reported the need for information about PPA, such as cognitive changes, communication difficulties and what to expect, some PwPPA report an uncertainty in receiving information early on (Davies et al. 2025). Similar findings were reported in Davies et al. (2024) study about family members' needs. One family member described how it was difficult to raise the topic of future planning with their family member with PPA. While future planning was reported to be a difficult topic by one family member in the study, overall, family members reported the need to address this issue (Davies et al. 2024). Future planning has also been identified by speech and language therapists (SLTs) as an area of need, extending beyond planning for future changes in communication. Davies et al. (2025) found that SLTs identified planning for the future as an area of need for both the PwPPA and family members, and that this needed to include planning for future services and end‐of‐life decisions. Davies et al. (2025) findings are in line with Volkmer et al. (2023) recommendations from expert SLT clinical researchers internationally, who recommended preparing for the future by discussing decisions about finances and healthcare, power of attorney and advance care planning (ACP), were identified as a best practice principle.

Both the Norwegian dementia guideline and the UK National Institute for Health and Care Excellence (NICE) dementia guideline advocate the importance of all healthcare professionals addressing future planning with their patients with dementia. The guidelines recommend that the opportunity to discuss ACP be offered to people living with dementia and their CPs (Helsedirektoratet 2017; NICE 2018b). The NICE guidelines on decision‐making and mental capacity are even more specific in their recommendations on ACP, and state that “Skilled practitioners need to be able to have sensitive conversations with people in the context of a trusting and collaborative relationship, and provide the person with clear and accessible information to help them make these important decisions.” (NICE 2018a, p. 14). Health and social care practitioners should adapt the conversation to the person with dementia and/or the caregiver's wishes and needs, and be aware that some people may feel uncomfortable or prefer not to talk about the topic (Helsedirektoratet 2017; NICE 2018a). The UK NICE guidelines on Decision‐making and mental capacity emphasise that all health and social care practitioners have a responsibility to help the person make an informed choice about taking part in developing an advance care plan, and if they want to enable them to take part (NICE 2018a). These guidelines build on legislation, which, among other things, defines the patients’ rights, such as the Mental Capacity Act in the UK (“Mental Capacity Act,” 2005), and the Patient and User Rights Act (“Lov om pasient‐ og brukerrettigheter ” 1999) in Norway. Whilst the guidance has extended the legislation by providing broad clinical recommendations, neither provides direction on how to operationalise a conversation on advanced decision‐making.

Applied Conversation Analysis (CA) is a method to systematically study social interaction. It has been applied to research in healthcare settings over the last 35 years, resulting in a substantial body of work (Barnes 2019). Applied CA allows us to study practical problems and dilemmas that healthcare professionals and patients encounter in clinical settings, the communicative practices they use to navigate these (Pino and Parry 2019b), and how clinical guidelines play out in practice (Barnes 2019). In one study, applied CA was used to explore how capacity assessments were conducted by healthcare professionals, including SLTs, with patients with acquired brain injury (Foulkes et al. 2024). In recent years, there has been a focus on end‐of‐life conversations in this field of research (Barnes 2019). Ekberg et al. (2021) conducted a rapid review of studies employing CA or discourse analysis to explore conversations about planning for end‐of‐life care and illness progression. Findings from the review were synthesised to provide five core recommendations for healthcare professionals having conversations on planning for end‐of‐life care and illness progression: (1) ascertain a patient or family member's perspective before offering your own; (2) where possible, mirror the language of the patient or family; (3) create opportunities to discuss the future; (4) be clear about uncertainty and (5) display sensitivity (Ekberg et al. 2021). These recommendations are in line with the NICE dementia and decision‐making and mental capacity guidelines (NICE 2018a, 2018b) and the Norwegian dementia guidelines (Helsedirektoratet 2017). Additionally, they expand on the guidelines by providing specific recommendations on how to manage the conversations.

Guidelines, such as the NICE dementia guidelines (NICE 2018b) state all health and social care professionals discuss ACP. However, recommendations for future care planning and end‐of‐life care may not be sufficient to support healthcare professionals in implementing these in everyday interactions. In their umbrella review of effectiveness and experiences of ACP for people living with dementia, Wendrich‐van Dael et al. (2020) analysed studies that included different healthcare professionals, such as general practitioners and nursing home staff. They found that lack of resources, including time, skills, and knowledge, was a barrier for healthcare professionals to engage in ACP conversations, whilst equipping professionals with the knowledge and communication skills necessary to engage in ACP was a facilitator (Wendrich‐van Dael et al. 2020). However, little is known about how SLTs can address ACP with people with dementia. One way of providing healthcare professionals with knowledge and communication skills is through protocolled intervention programmes that provide prompts and guides to facilitate such discussions.

In the Better Conversations with PPA (BCPPA), a manualized communication partner training (CPT) program usually delivered by a speech and language therapist to a person with PPA and their care partner, conversations on future planning are integral to the final session. Future planning was identified as an important topic to address during the coproduction of the intervention with PwPPA and their CPs, and is therefore woven into the intervention itself (Volkmer et al. 2021). The BCPPA intervention comprises four sessions, and each session follows a protocolised step‐by‐step session plan. Tailored handouts with information are provided to the dyad in each session. The fourth session focuses on problem‐solving and planning for future changes in communication (Volkmer et al. 2024). Dyads are provided a handout with information about further therapy, support networks, modifying communication strategies, re‐accessing speech and language therapy services, and changes in decision‐making capacity. Research on PPA to date has shown promising improvements in conversation, communication‐related confidence, and quality of life following the intervention (Volkmer et al. 2023). Cultural adaptations to Norwegian (Winsnes et al. 2025) and other languages are currently underway. Participants have provided overwhelmingly positive feedback about the intervention overall (Volkmer et al. 2023; Winsnes et al. 2025), however, this study aimed to analyse objectively, using Applied CA, how the topic of planning for the future in BCPPA is undertaken in conversations during session four of the BCPPA intervention.

## 2 Method

### 2.1 Design

In this study, we used Applied CA to explore video‐recorded intervention sessions from the BCPPA intervention, where the topic of future planning is included in the session plan protocol (session four). The data were recorded as part of a study piloting the Norwegian version of the BCPPA, and results on acceptability and feasibility can be found in Winsnes et al. (2025).

### 2.2 Ethics

The current study was approved by the Norwegian Agency for Shared Services in Education and Research (reference number 147255). It complies with the Declaration of Helsinki and the National Guidelines for Research Ethics in the Social Sciences and the Humanities. Written informed consent was obtained from the participants (PwPPA and CPs) before enrolment in the study. Pseudonyms are used for all participants, and people and places mentioned in the data.

### 2.3 Sampling and Recruitment

Participants in the study were recruited as part of an intervention study exploring the cultural adaptation of BCPPA to Norwegian (Winsnes et al. 2025), which was reported in line with the Framework for Reporting Adaptations and Modifications‐Enhanced (Wiltsey Stirman et al. 2019). Recruitment took place between February 2023 and January 2024. PwPPA were recruited from the University Hospitals Memory Clinic, a geriatric outpatient clinic at a local hospital, private speech and language therapy services, and adult educational centres providing speech and language therapy. PwPPA were eligible to participate if they could give informed consent and were able to receive the treatment in Norwegian. Potential participants were provided with written information about the project by their treating medical doctor or SLT; if they agreed, their contact information was shared with first author IW, who then contacted them with further details about the intervention study. The CPs in the project were recruited through the PwPPA, who were asked to identify a person (family or friend) whom they talked to regularly.

### 2.4 Data Characteristics

Four dyads (PwPPA and CPs) consented to participate. Table 1 provides an overview of the dyads. In addition to the dyads, the first author, IW, is the treating SLT in all the recordings. IW is a trained SLT with several years of clinical experience. She did not have previous experience with the approach or specific training in conversations about future planning. However, she received a full day of training, alongside ongoing supervision, in the BCPPA approach before the intervention started.

**TABLE 1:** Overview of the participants' characteristics.

Participants	Relationship	Diagnosis (PwPPA)	Time post‐diagnosis (years)
Dyad 1	Married	Mixed PPA	< 1
Dyad 2	Married	Mixed PPA	< 1
Dyad 3	Married	nfvPPA	< 1
Dyad 4	Married	nfvPPA	< 1

Table 2 provides an overview of language and cognitive function at the time of inclusion in the study, as measured with the Comprehensive Aphasia Test‐ Norwegian (Swinburn et al. 2021) and the Mini‐Mental State Examination—Norwegian Revised (Strobel and Engedal 2021).

**TABLE 2:** Overview of language and cognitive function at inclusion.

	PPA1	PPA2	PPA3	PPA4
**CAT‐N**				
*Auditory comprehension (66)*	62	64	59	59
*Reading comprehension (62)*	57	53	52	56
* Repetition (74)*	66	66	59	53
* Naming (79)*	28	71	63	57
*Oral reading (70)*	70	69	70	70
**MMSE‐NR3**				
*Orientation (10)*	6	9	8	9
*Immediate recall (3)*	3	3	3	3
* Calculation (5)*	5	1	5	5
* Delayed recall (3)*	1	2	3	2
*Language and praxis (8)*	5	8	8	8
* Figure copying (1)*	1	1	1	1
Total MMSE Score (Total possible score = 30)	21	24	28	28

Abbreviations: CAT‐N, Comprehensive Aphasia Test‐ Norwegian; MMSE‐NR3, Mini‐Mental State Examination—Norwegian Revised.

The recordings analysed in this study were taken from the fourth session of the BCPPA, which is the last session (we refer the reader to Volkmer et al. (2021) for a detailed description of the intervention development and outline of all four sessions, and Winsnes et al. (2025) for details of the Norwegian adaptation). The initial three sessions provided the SLT with time to get to know the participants and build a trusting relationship before addressing future changes in the last sessions. Additionally, the topic of future changes was introduced at the end of the fourth session. This, the fourth session, begins with reviewing home‐based tasks and discussing one or two things the participants remember from the last session, before moving on to practising strategy use and reviewing the treatment goals set in a previous session. After this, future changes in communication are introduced as a topic. The BCPPA manual does not detail how to address this, rather it lists topics to address (see Table 4).

We collected data from Dyads 1, 2 and 3 in the Socio‐Cognitive Laboratory at the University of Oslo. The delivery of the intervention and recording of this were done in the laboratory. We used a room decorated to resemble rooms used in ordinary clinical practice, and the control room for the recording device was in a separate room. Dyad 4 was unable to travel to the university; therefore, the SLT travelled to the participant's home to deliver the intervention there. The intervention was delivered in their living room, and only the dyad and SLT were present. Viso, a secure video recording app were used to record the sessions with a smartphone. See Table 3 for an overview of data characteristics.

**TABLE 3:** Overview of data characteristics.

Participants	Place	Recording device	Total length of BCPPA session four recordings
Dyad 1	The Socio‐Cognitive Laboratory, the University of Oslo	PTZ Full HD camera and microphone hanging from the ceiling	1 h 4 min.
Dyad 2	The Socio‐Cognitive Laboratory, the University of Oslo	PTZ Full HD camera and microphone hanging from the ceiling	45.23 min.
Dyad 3	The Socio‐Cognitive Laboratory, the University of Oslo	PTZ Full HD camera and microphone hanging from the ceiling	36.08 min.
Dyad 4	In the participant's home	Viso application on a smartphone.	47.24 min.

### 2.5 Analysis Procedure

Using Applied CA allowed us to describe and analyse talk‐in‐interaction in this study. Applied CA has been highlighted as the “gold standard” for analysing recorded clinical communication (Ekberg et al. 2021). CA involves collecting sound or video recordings of naturally occurring talk that are then transcribed and analysed, where transcribing data is part of the analysis process (Sidnell 2010, pp. 20–29). We position our work within the Applied CA field, and have conducted “motivated” looking (Barnes 2024). That is, even though we did not set out to identify a specific phenomenon, our work was undertaken to describe how planning for future changes in communication is addressed in the BCPPA intervention. Thus, authors MH and EK, who did the initial transcribing and analysis under the supervision of IW, watched the video recordings from each of the dyads' fourth session in the BCPPA intervention, and orthographically transcribed the recordings from where the overarching topic planning for future change in communication was introduced. Table 4 provides an overview of session four, with the three main activities in the session, the topics that might be addressed during discussions about the future, and in which extracts the different parts are analysed.

**TABLE 4:** Overview of session 4.

Session 4	Extract no.
1. Role play or record the couple in the session, practising strategy use	
2. Review goals set in session 2	Extract 3
3. Discuss future changes in communication	
*Further therapy*	Extract 1
*Support networks*	
*Modifying communication strategies*	
*Re‐accessing SLT services*	
*Changes in decision‐making capacity*	Extracts 2 and 5

*Note*: text in italic indicates topics addressed within the third main activity.

ELAN software was used for segmenting the talk and manually transcribing the data (ELAN (Version 7.0) [Computer software] 2025). During the initial analysis, patterns in the data were observed, and a collection of identified phenomena was made. For example, when the SLT produced a first pair part, where she provided information about the Dyads SLT, and the second pair part was a receipt from the PwPPA or CP. This collection of sequences was then transcribed in more detail by MH, EK, and IW, using the Jeffersonian transcription system (Jefferson 2004). In the extracts, we mainly use a two‐linear transcription with Norwegian on the first line and idiomatic English translation on the second line. Where visual conduct is transcribed, a third line containing visual conduct as a comment from the transcriber is added. Despite the participants utilising a wide range of multimodal resources (e.g., gestures and writing key words), visual conduct was transcribed selectively, which is in line with recommendations for CA transcription (Oloff and Hepburn 2024). Our findings were analysed in relation to the five core recommendations for conversations about planning for end‐of‐life care and illness progression identified by Ekberg et al. (2021), which align with the NICE dementia and NICE decision‐making and mental capacity guidelines (NICE 2018a, 2018b) and the Norwegian dementia guidelines (Helsedirektoratet 2017).

**FIGURE 1:**
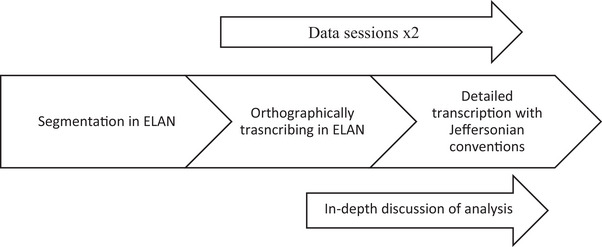
Overview of analysis process.

As IW is the SLT in the recordings, it has been considered necessary that MH and EK did the initial transcribing and analysis to increase the trustworthiness of the data. To further enhance the trustworthiness of the data, we brought it to two data sessions with experienced CA researchers. Showing data in data sessions is a core research practice in CA, as the data are collectively and critically analysed to enhance rigor of analysis (Betz 2024). Finally, IW and AV discussed all the analyses in depth. Figure 1 shows an overview of the analysis process.

## 3 Results

In these results, we present how the topic ‘future changes in communication’ is opened, and how options and actions are sensitively negotiated in relation to this topic using hypothetical scenarios. We start by presenting the opening of the topic, before we show how the SLT introduces the topic of “changes in decision‐making capacity”. Finally, we present a case that deviates from the others. In this case, the care partner pursues the topic of illness progression before changes in decision‐making capacity are introduced by the SLT.

### 3.1 Opening Conversations about ‘the Future’

The introduction to the topic of—future changes in communication—and planning for these follows the same sequential structure in all four dyads. As in Foulkes et al. (2024), the opening phase is brief. The SLT opens the conversation about “the future” by explicitly mentioning the future, then she addresses further speech therapy, either by mentioning the name of the SLT who is going to follow up or referring to the SLT they already have. She also provides the dyad with the handout for the session and points to it, not visible to the camera, but presumably to the name of the SLT.

Extract 1 provides a typical example of an opening of the topic.

**FIGURE 2:**
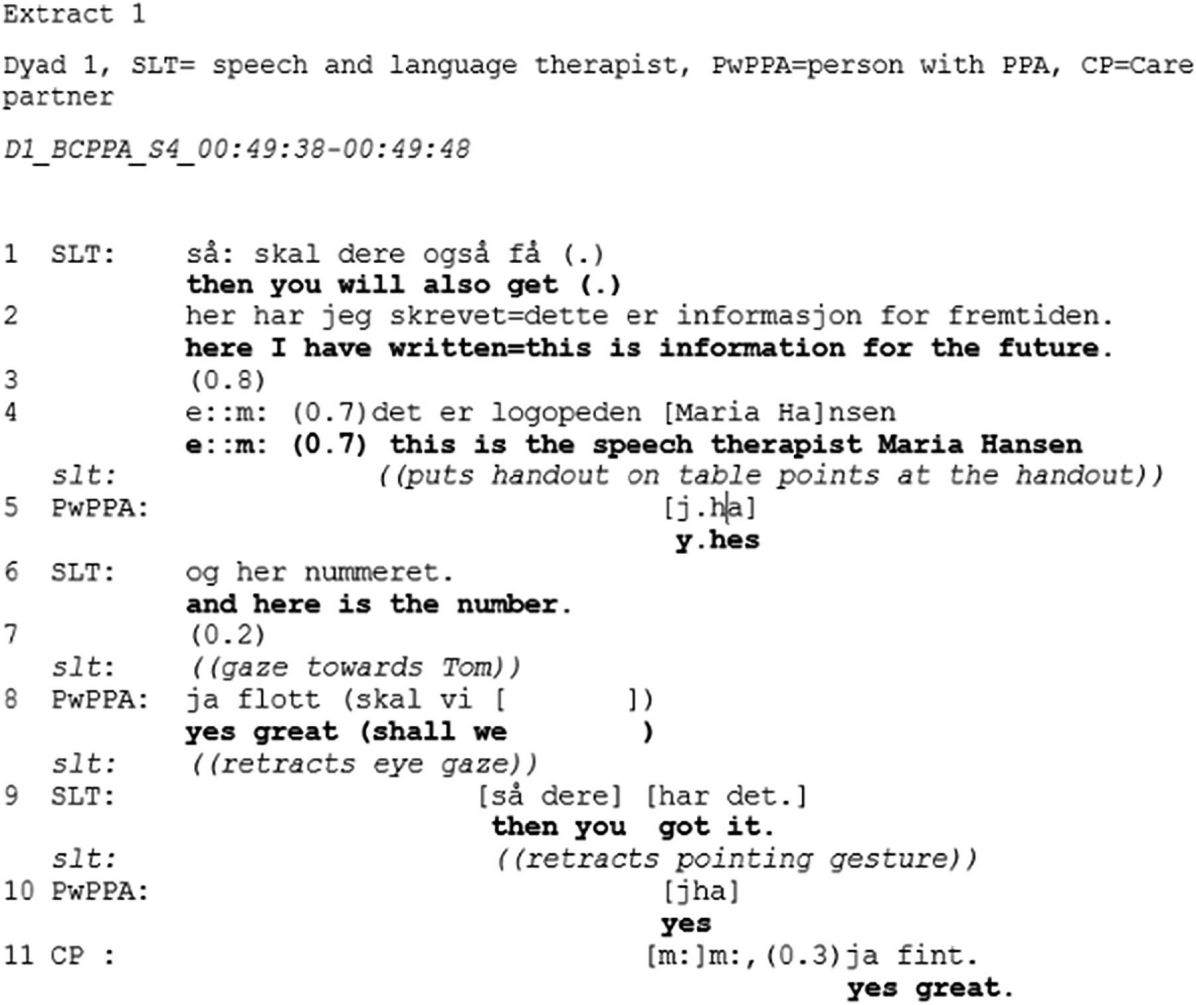
Extract 1.

Extract 1 begins with the SLT introducing information about the future by producing a turn (lines 1–2) “*then you will also get*” before she cuts off and produces “*here I have written this is information for the future*”. Here, the SLT mentions the future, without mentioning possible changes, creating an opportunity for the PwPPA and CP to continue the topic without imposing any particular direction on the conversation. In doing this, she demonstrates sensitivity and creates an opening for the dyad to provide their opinion on the topic before offering her own. Previous research has shown how healthcare professionals orient to disease progression as a sensitive topic, and gradually prepare the patient and care partner to address topics, such as ACP and prognosis, by alluding to them (Parry 2024). Whilst producing “*this is*”, she puts a handout containing information about the future on the table in front of them. She then produces “*this is the speech and language therapist Maria Hansen*” (line 4), whilst at the same time pointing at the handout. Whilst still pointing at the handout, the SLT produces “*and here is the number*” (line 6). During the consequent gap (line 7), the SLT gazes at the CP. Gaze may function as a turn allocation component (e.g., next speaker selection) (Auer 2021), thereby indicating that a response to the provision of the name and number is expected. In line 8, the CP receipts the invitation, producing a preferred response, “*yes great*”, without expanding on the topic. The SLT produces a turn containing a declarative sentence “*then you got it*” (line 9) and at the end of her turn in line 9 retracts the pointing gesture. The sequence is closed when the CP and PwPPA receipts the SLT's turn by producing “*yes*” and “*yes great*”. In this sequence, CP and PwPPA do not take up the opportunity to expand on the topic of the future. And in line with the guidelines, the SLT displays sensitivity by not pursuing the topic any further at this moment, but rather waiting until later in the session to revisit it (Helsedirektoratet 2017; NICE 2018a).

### 3.2 Sensitive Exploration of Options for the Future

As part of the overarching topic, *future planning*, changes in decision‐making capacity are also addressed later in the session (see Table 3). This topic is introduced near the end of the session. The introduction of this topic follows the same sequential structure in all four dyads, with the SLT opening the more specific topic of changes in decision‐making capacity, providing an account for raising this topic, before lasting power of attorney (LPA) is raised as an option either by the SLT or the dyad.[Fig jlcd70235-fig-0003]

**FIGURE 3 jlcd70235-fig-0003:**
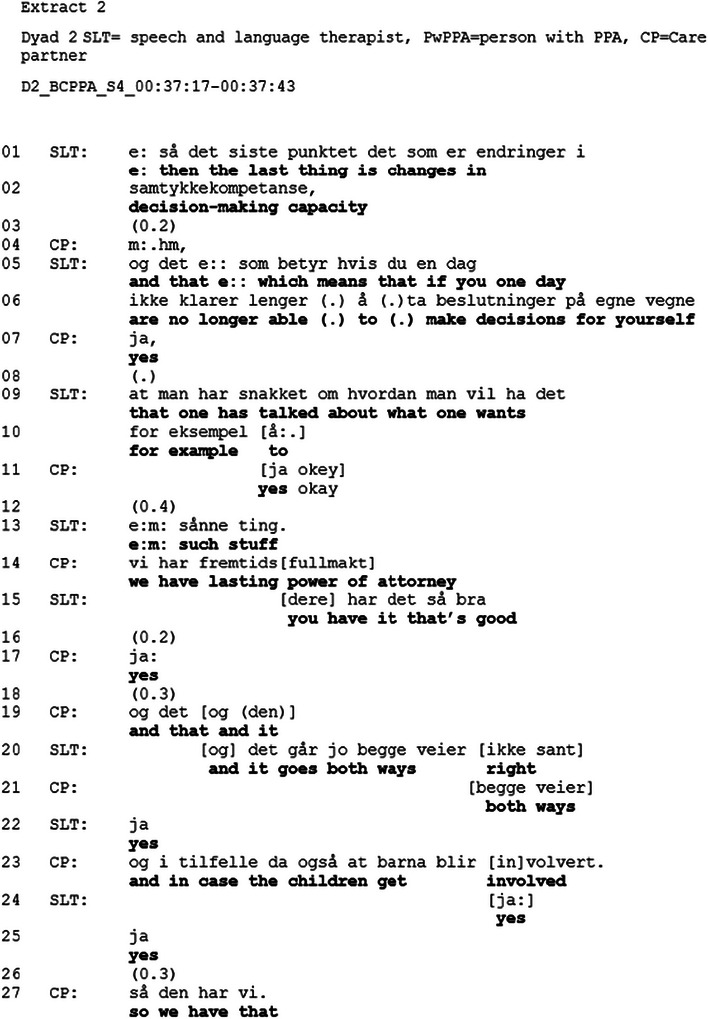
Extract 2.

In the extract above, the SLT opens the topic of changes in decision‐making capacity by pointing to the handout and producing “*and the last topic is changes in decision‐making capacity*”. The CP produces a continuer in line 1 before the SLT in lines 5–6 begins to produce an account for why she is initiating this topic. The account is formulated as a hypothetical scenario “*if you one day are no longer able to make decisions for yourself*”. Hypothetical scenarios have been shown to promote discussion about illness progression when the patient or family members have shown reticence to discuss the topic (Ekberg et al. 2021). In line 6, the SLT produces “du” (you 2SG), indicating that the turn is addressed to the PwPPA. However, it is the CP who responds to the turn by producing “*yes*”, with a slightly rising final intonation (line 7), indicating that he is handing the floor back to the SLT (i.e., “yes” functions as a continuer) (Gardner 2001, pp. 129–130). Following this approval to continue talking on the topic, the SLT then produces another account for why it is important to address changes in decision‐making capacity, “*that one has talked about what one wants for example*” (lines 9–10). The turn is prefaced with reference to the personal pronoun “one” in the general sense, by using “*that one*” and referring to a hypothetical scenario by producing a turn‐final “*for example*”. These strategies create opportunities to discuss the future, but depersonalise the topic, thus making provision for any reluctance the patient or family member may feel in discussing the topic in relation to themselves (Ekberg et al. 2021). In line 15, the CP uses the personal pronoun ‘we do’, bringing the action “*we do have lasting power of attorney*” into their personal sphere of control. The SLT responds to this (line 16) turn partly in overlap with CP, acknowledging that they have taken action by using the personal pronoun “you”. From lines 20 to 24, Walter provides more information about the detailed action included in the LPA before producing a formulation, *“so we have that”* (line 27), which sums up the gist of the sequence (i.e. importance of having prepared for future change) and closes the sequence (Heritage and Watson 1979).

### 3.3 A Deviant Case

In the following, we will show a case that deviates from the three others in our study. We will first show how the topic of the future is introduced by the SLT, as a response to a question from the dyad, and how the dyad pursues the topic of the future by asking questions about the progression of the illness. We will then turn to how the topic of future changes in decision‐making capacity is treated by the same dyad.

#### 3.3.1 Care Partner's Use of Impersonal Pronouns in Pursuit of Prognostic Information for the Future

The two following extracts are from the same sequence. The entire sequence starts at the point when the participants are reviewing goals set in the intervention and spans 251 lines. The line numbers on the following extracts show how far through the sequence the opening occurs. Extract 3 is part of a longer sequence, where they review the goals set in a previous session. They have talked about how a strategy may be helpful for some people but not for others. In this extract, we will focus on lines 189–190, where the SLT opens the topic of the future.

**FIGURE 4:**
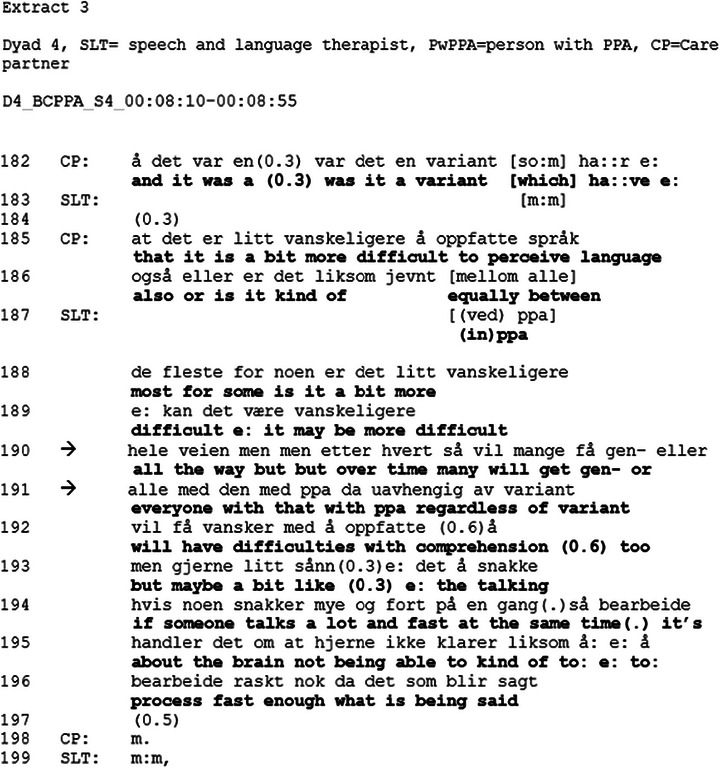
Extract 3.

The extract begins with the CP starting a turn (line 182), and reformulating it as a question about PPA, “*and it was a was it a variant which have that it is a bit more difficult to perceive language also or is it kind of equally* between”. In this turn, the CP demonstrates her knowledge about PPA, which may indicate readiness to address future changes (Pino and Parry 2019a). By using a third‐person singular personal pronoun “it”, which has been found to be a way of implicitly talking about end of life (Ekberg et al. 2019; Pino and Parry 2019b), the CP distances herself, reducing the personal connection. In overlap with the CP, the SLT begins to produce an answer about the nature of PPA at line 190 and in line 191, opens up the topic of the future by stating “*over time*” when she starts describing how PPA can change over time. In this description, at line 191, the SLT uses the indefinite pronoun “*everyone*” with PPA progression. By referring to all PwPPA, she orients to this in general terms, thus allowing the CP and the PwPPA to apply this to their own situation without forcing them to (Ekberg et al. 2021). Using this type of general case formulation, instead of patient‐specific pronouns, has been found to soften the direct relevance to the specific patient (Land et al. 2019). In line with Ekberg et al. (2021) recommendation about mirroring the language of the patient, the SLT carefully adapts her talk to mirror the distance created by the CP's pronoun use.

Following a longer gap (7.0) where the goal review has continued, the CP re‐opens the discussion on the future.[Fig jlcd70235-fig-0005]

**FIGURE 5 jlcd70235-fig-0005:**
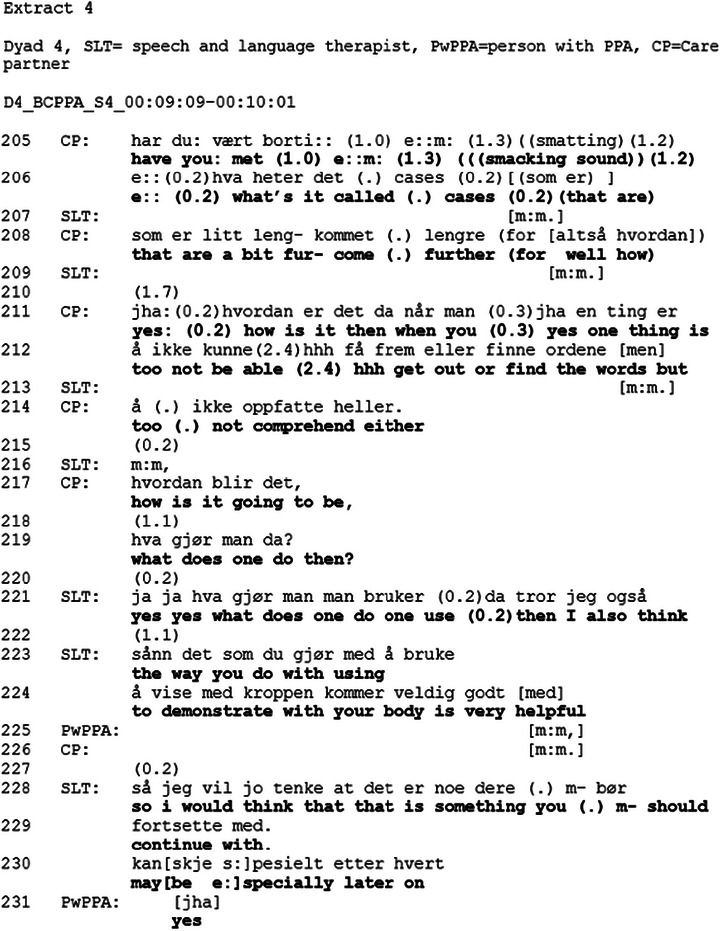
Extract 4.

The extract begins with the CP producing a turn (lines 205–214) containing several pauses. These pauses may indicate that this is a delicate topic for Sarah (Silverman and Peräkylä 1990). In lines 207, 209 and 213, the SLT produces continuers in overlap with Sarah, which function as an encouragement for Sarah to continue (Parry et al. 2014). Similar to the use of “it” in Extract 3, the CP formulates her turn in general terms, by referring to other “cases” the SLT may have met in line 206. She asks “*how is it going to be*” in line 217, again indicating that she does not have a strong personal connection to ‘it’. At line 219, the CP uses the impersonal pronoun “one” in her question “*what does one do then*”, further reinforcing the general terms of her questions. The SLT continues the careful mirroring (Ekberg et al. 2021) and responds to the CP's turn by repeating “*what does one do then*”. Only then, when answering her own repetition of the question, does the SLT use “*I also think*” (line 221) to qualify her response, thus treating the topic with ongoing delicacy as previously seen in conversations around prognosticating (Anderson et al. 2020). She relates her answer (lines 221–230) directly to the PwPPA and CP, using the personal pronoun “you” and emphasising that they should continue with what they are doing (i.e. body language), while not directly stating that the PwPPA's auditory comprehension will decline.

#### 3.3.2 Sensitively Navigating Future Decision‐Making and Requests for Hypotheticals

We will then show how the topic change to future decision‐making capacity is treated. In extract 5, the introduction of cision‐making capacity follows the same sequential structure as in the other dyads, as illustrated by extract 2. However, in contrast to Dyads 1, 2 and 3, where the sequence is closed after information about changes in decision‐making capacity is provided, Sarah, the close other, produces a question about the future.[Fig jlcd70235-fig-0006]

FIGURE 6Extract 5.

**Figure:**
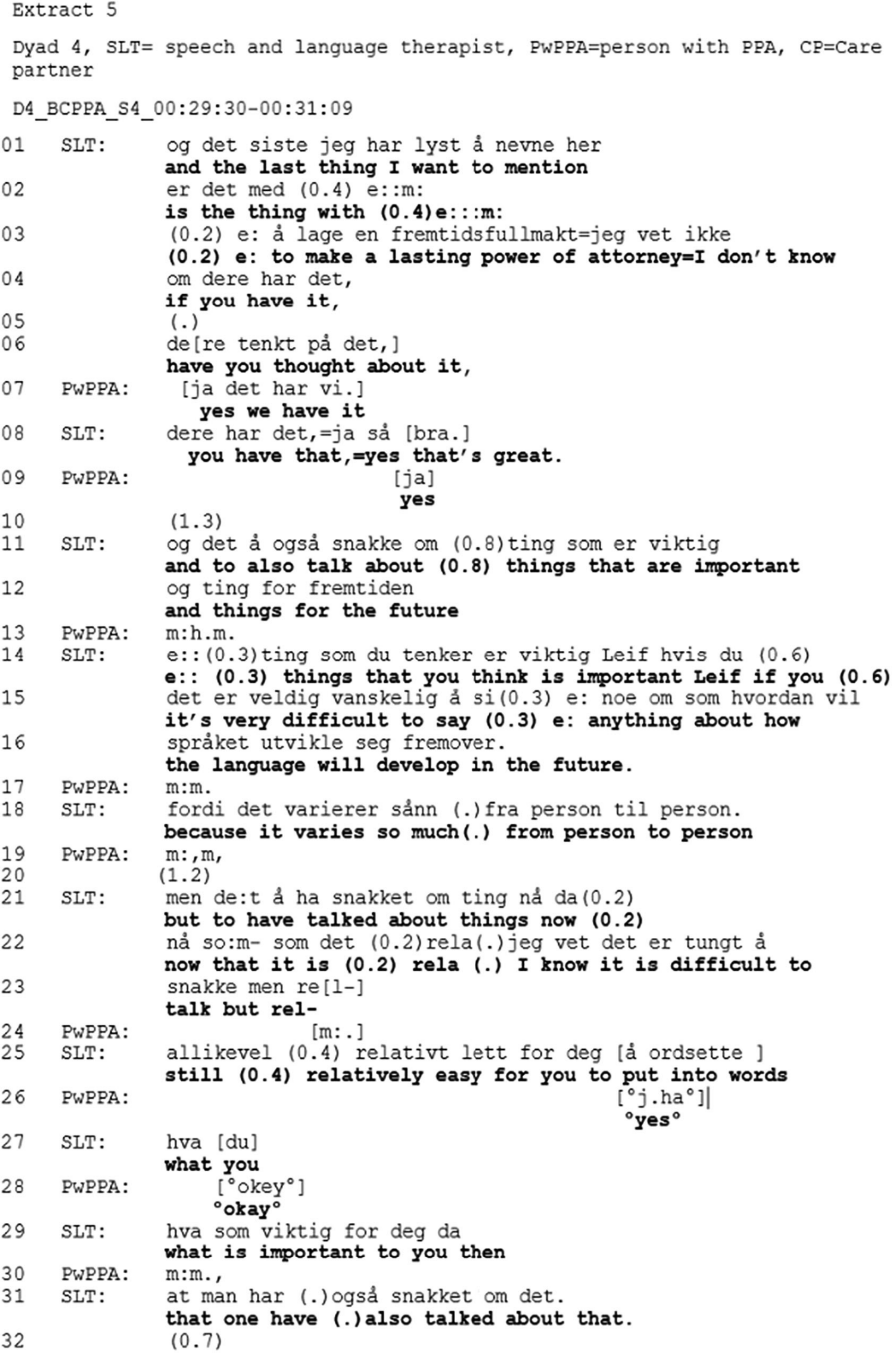

**Figure:**
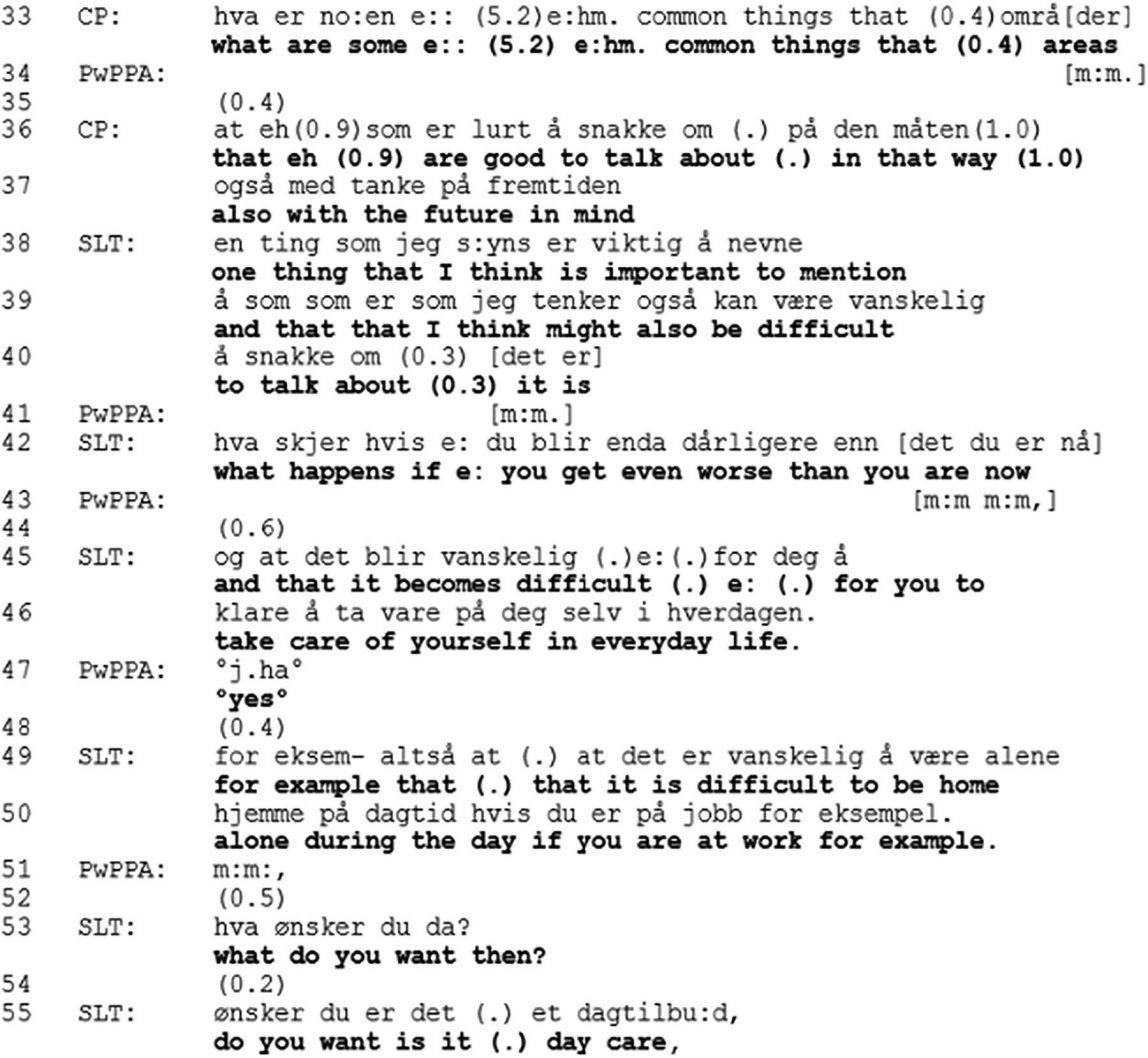

In Extract 5, lines 1–7, the topic of changes in decision‐making capacity is introduced in the same way as to the other dyads (illustrated in Extract 2). After the topic is introduced by the SLT, the PwPPA responds in overlap with the SLT's turn, confirming that they do have an LPA. The SLT provides an approving evaluation, “*you have that yes that's great*” (line 9), which PwPPA receipts with “*yes*” at line 10. After a pause in line 11, the SLT self‐selects to talk and indicates an extension of the topic (line 12) with “*and*” followed by further suggestions that they discuss possible future decisions, over lines 12–32. Rather than explicitly stating that this should happen before his language progresses, the SLT hedges their prognosis by indicating “*it's very difficult to say anything about how the language will develop in the future*” (lines 16–17), and by doing so, displays prognostic uncertainty (Anderson et al. 2020). To be clear about the uncertainty about the future (e.g., timeframe of progression) is one of the core recommendations from Ekberg et al. (2021). Whilst the PwPPA produces minimal responses (e.g., mm in line 20) to the SLT's account, there is another pause in talk at line 33, before the CP self‐selects to talk. She produces a question, asking which are common future decisions that are discussed, “*what are some common things that areas*”. The SLT produces a minimal response (mm) in line 34 in overlap with CP's turn, indicating that she is listening and encouraging the CP to continue. After a short pause, the CP completes the turn, “*that which we should talk about in that way also with the future in mind*”. The pauses and hesitancy signal the delicacy of the topic (Silverman and Peräkylä 1990). The CP's question is framed in general terms, asking for topics that, in general, are important to discuss for people in their situation. This way of framing the question allows the SLT to provide hypothetical scenarios, which in turn allows them to discuss the future without having to commit to accurate prognostication (Parry et al. 2014). The SLT's turn in lines 40–41 contains a parenthetical sequence which qualifies the continued delicacy of the topic “*which I think might be difficult to talk about*”. The SLT produces a hypothetical question, “*what happens if you get even worse than you are now”*, in line 43. She then continues with two hypothetical scenarios in lines 46–47 and 50–51. The PwPPA produces a continuer in line 52, before the SLT produce a new hypothetical question, and after a pause, she produces a suggestion to the question in the form of an option. After Extract 5, the SLT continue to list different options (e.g., daycare centre for people with young‐onset dementia, home care). Option‐listing may be a way of facilitating active participation from the PwPPA in decisions about the future. Foulkes et al. (2024) found that healthcare professionals used option‐listing to facilitate decision‐making by the patients. The topic of future changes is revisited one more time in the session. It is the CP who reintroduces the topic by asking for prognostication, and similarly to Extract 5, uses “it” and “that” instead of “progression” or “death”. In the same way as in Extract 5, the SLT displays prognostic uncertainty in her answer (Anderson et al. 2020). After this, the SLT lists more options for the future, before the session moved on to other topics.

Hypothetical scenario sequences often require considerable interactional work, and they may achieve multiple goals: they give the patient and care partner a realistic understanding of the disease and prognosis to help plan for the future, whilst also being sensitive to their preferences and not being too pessimistic (Land et al. 2019). Using hypothetical questions and scenarios is one way to create opportunities to discuss the future, and is one of Ekberg et al. (2021) five core recommendations.

## 4 Discussion

Using applied CA, this study explored how the SLT and PwPPA and their CPs participating in the BCPPA intervention study treated the topic of future planning. The sequential stepwise introduction to future planning observed in the video‐recorded sessions followed the BCPPA session plan protocol. The interactional practices we identified are summarised in Table 5.

**TABLE 5:** Overview of interactional practices that promote sensitive conversations about the future.

Interactional practice	Description
Hypothetical future scenario.	Referring to future changes as a hypothetical scenario using formulations such as “if you get worse”.
General case formulation	Refer to future changes using a general formulation, i.e., “one might experience”, thereby allowing the dyad to choose if they want to relate it to themselves or not.
Acknowledging prognostic uncertainty.	Acknowledge the uncertainty surrounding the timeframe for illness progression.

The structure of the BCPPA allows for a gradual introduction to the topic, starting with a discussion on future communication changes and the need for further speech and language therapy, as shown in Extract 1, where the SLT introduces the future by referencing the handout and stating that it is information for the future, before she provides information about further speech and language therapy. By mentioning the future, without stating her own opinion, the SLT creates an opportunity for the PwPPA and CPs to provide their perspective on the future without imposing her own view (Ekberg et al. 2021). Following the BCPPA session plan protocol, exploring future changes in decision‐making and options related to maintaining a voice in future decision‐making are introduced after several topics related to the future (see Table 3) have been addressed. Conversations about the future in the context of a progressive disease may be viewed as a delicate topic (Silverman and Peräkylä 1990). By gradually introducing the topic, the SLT, as recommended by research on conversations about illness progression and clinical guidelines on dementia, displays sensitivity (Ekberg et al. 2021; NICE 2018a). Extract 2 provides an example of how the SLT opens the discussion on changes in decision‐making capacity by referencing the handout and producing an account that is formulated as a hypothetical scenario. It may be that by referencing the handout, the topic is treated as something that is mentioned to everyone, something general. The generality is also observed in the pronoun use, where the SLT use a general case formulation instead of a personal pronoun (Land et al. 2019). This creates an opportunity to discuss the future without forcing the PwPPA or the CP to talk about it, which is in line with the third recommendation (i.e., create opportunities to discuss the future) from Ekberg et al. 2021) review. Foulkes et al. (2024) found that healthcare professionals provided information as part of a preparation phase to inform the patient's decision‐making. It might be that the account provided by the SLT in the current study functions as a way of preparing the dyads for making decisions about the future. By introducing the topic of future planning gradually and referencing the handout, the SLT provides the PwPPA and the CP the opportunity to treat the topic as merely information from the SLT, which does not require them to pursue the topic further. This approach is in line with the Norwegian dementia guidelines and the NICE guidelines on Decision‐making and mental capacity, which recognise that not all people may want or may not yet be ready to talk about the future (Helsedirektoratet 2017; NICE 2018a). Indeed, most dyads do not engage in the topic, despite several opportunities to discuss it, and the discussion on the topic is postponed. Similar to Foulkes et al. (2024), the data (see Extract 2 for an example) from Dyads 1,2, and 3 showed that the closing sequence contained a plan for future action (e.g., “we have an LPA which states that…” or “we will create an LPA”)

In contrast, the CP from Dyad 4 in extracts 3, 4 and 5 demonstrates readiness to discuss prognosis and future planning in her pursuit of information and hypotheticals. In Extract 3, lines 182–186, she asks about impairment in auditory comprehension in PwPPA. In her turn, she demonstrates knowledge about what she already knows. In a study about life‐expectancy estimates, Pino and Parry (2019a) found that patients or a co‐present companion may share what they already know, as a way of orienting to the delicacy of the topic, demonstrating readiness to address it, and preparing the conversation environment for delivery of estimates. In the current study, the CP's turn may function as a way of showing readiness for the topic. The next turn does two things; firstly, the SLT formulates her answer in general terms, thus providing the opportunity for the dyad to relate this to their situation, but without forcing them to, using general case formulations mirroring the CP's pronoun use (Land et al. 2019). Secondly, she orients to the future by producing “over time”, thereby creating an opportunity to discuss future changes (Ekberg et al. 2021). In the next extract (Extract 4), which begins shortly after Extract 3, the CP addresses the topic of future changes in communication. This Extract highlights the interactional work that is required when addressing delicate topics. The CP's turn contains several pauses and hesitations, whilst the SLT encourage her to continue by producing continuers (Parry et al. 2014; Silverman and Peräkylä 1990). In these two extracts, the CP demonstrates readiness to discuss future planning. However, it is oriented to as a delicate topic and addressed using general terms and hypothetical scenarios. Thereby softening the direct relevance of the topic and creating opportunities to discuss the topic in a way that makes it possible for the participants to regulate how much they relate this to themselves (Ekberg et al. 2021; Land et al. 2019).

When changes in decision‐making capacity are introduced to Dyad 4, illness progression has already been addressed previously in the conversation. Still, the topic is introduced following the same sequential structure as with the other dyads. However, instead of topic closing after LPA is mentioned, the SLT addresses illness progression, emphasising prognostic uncertainty (Anderson et al. 2020). Which is in line with Ekberg et al. (2021) who recommend acknowledging the uncertainty around illness progression, such as the timeframe for the progression. In the extract, the SLT displays sensitivity, which has been highlighted as important (Ekberg et al. 2021; NICE 2018a), by producing a parenthetical sequence (lines 40–41), where she acknowledges that the topic may be difficult to discuss. As demonstrated in Extracts 3 and 4, the participants have done interactional work to prepare the conversation environment for discussions about illness progression, which may be the reason Dyad 4 pursued the topic further.

Illness progression is discussed using general case formulations, and hypothetical questions and scenarios. This allows the PwPPA and the CP to engage with the topic without having to agree that this is how their future transpires, and it has been highlighted that this is particularly useful when the trajectory of the disease is uncertain (Ekberg et al. 2021; Parry et al. 2014). By framing the option‐listing (e.g., “if you get worse… one option is a day care centre, another home care”) as hypotheticals, the SLT facilitates active involvement in decisions about the future by the PwPPA (Foulkes et al. 2024) without forcing the PwPPA and CP to relate this hypothetical future to themselves. At the same time, the PwPPA and CP are provided with information which might help them with decision‐making in the future (Land et al. 2019). Using hypothetical scenarios often involves substantial interactional work (Land et al. 2019). Dyad 4 have already indicated their readiness to discuss the topic, as shown in Extracts 3 and 4. The CP, when producing questions, uses general case formulation, which is mirrored by the SLT in her answer. The SLT uses hypothetical scenarios (e.g. *if you get worse*) to facilitate a conversation about future planning. Hypothetical scenarios have been highlighted as a sensitive and effective way to identify and prepare for patients’ future needs (Land et al. 2019), and by using them, the SLT creates opportunities to discuss the future, whilst displaying sensitivity (Ekberg et al. 2021).

### 4.1 Clinical Implications

This study highlights that with a protocolised checklist, such as that included in the BCPPA session plans, SLTs can provide sensitive and delicately negotiated opportunities to PwPPA to discuss and plan for their future care and support needs. Hypothetical scenarios may be a useful communication tool to support PwPPA to access conversations on the topic of future planning. Importantly, having undertaken several therapy sessions prior to the session on future planning, SLTs are in a position where they can have “sensitive conversations with people in the context of a trusting and collaborative relationship, and provide the person with clear and accessible information to help them make these important decisions” (Rec. 1.1.30, NICE guidance, 2018). Both Dementia and decision‐making guidance have highlighted that future planning is the responsibility of all health and social care practitioners working with people with progressive diseases (Ekberg et al. 2021; NICE 2018a). The BCPPA protocolised session plans promote future care planning as a routine and standard component of speech and language therapy care in line with previous research that has advocated for training and resources to help healthcare professionals engage in conversations about future care (Wendrich‐van Dael et al. 2020). Including these protocols in other dementia interventions may be one way for healthcare professionals to embed these conversations in ordinary clinical practice.

### 4.2 Limitations and Future Direction

The data in the current study were collected in a research setting, in Norway, and with lead author IW, an experienced SLT delivering the treatment. Despite taking precautions by engaging MH, EK, and AV in the analysis, and showing the data *(recordings and transcriptions of BCPPA sessions)* in data sessions, having the same person delivering the treatment and analysing the data is a limitation which should be addressed in future research. The study demonstrated how one experienced SLT negotiated the topic of future illness progression and future planning with four dyads. However, other SLTs might do it differently. Despite aiming to achieve a sample with diverse backgrounds, we only managed to recruit PwPPA less than a year post‐diagnosis. Further research should aim to include a larger and more diverse sample, including people with logopenic and semantic PPA. Collecting data from ordinary clinical practice, in different settings and across countries, would also be useful to better understand the nature of ACP conversations in BCPPA. Additionally, exploring the deontic and epistemic rights of the PwPPA should be analysed in future research. Despite not targeting dementia specifically, Ekberg et al. (2021) recommendations for communicating with patients and family members about illness progression and end‐of‐life care may be useful for SLTs working with PwPPA and their CPs, and should be addressed in future research. This might be done using the BCPPA protocol, as it comprises the elements necessary to address illness progression and end‐of‐life care. Thus, making it easy to incorporate the recommendations into a training package for SLTs.

## 5 Conclusion

This study provided preliminary evidence for the BCPPA as a useful resource when addressing future planning with PwPPA and their CPs. The structure of the BCPPA ensures that all participants receive information about future planning, and opportunities for addressing future illness progression and future planning are created several times. Depending on the participants' preferences, the topic of future changes in communication and ACP might be expanded on and discussed in more detail. In line with previous research, using hypotheticals seems to be a powerful strategy to address this delicate topic sensitively. Overall, the structure of the BCPPA allows for sensitive conversations about the future to inform decision‐making. The BCPPA has the potential to be a useful tool for SLTs working with patients with degenerative diseases, such as dementia, and future research should address this.

## Conflicts of Interest

The authors report there are no competing interests to declare.

## Ethics Statement

The current study was approved by the Norwegian Agency for Shared Services in Education and Research (reference number 147255).

## Consent

Written informed consent was obtained before enrolment in the study.

## Data Availability

The data analysed during the current study are not publicly available due to ethical restrictions related to the sharing of video data.
